# Effective palliative radiotherapy in primary malignant melanoma of the esophagus: a case report

**DOI:** 10.4076/1757-1626-2-6928

**Published:** 2009-08-19

**Authors:** Takeshi Nonoshita, Yoshiyuki Shioyama, Satoshi Nomoto, Saiji Ohga, Kayoko Ohnishi, Kazushige Atsumi, Kotaro Terashima, Shuji Matsuura, Katsumasa Nakamura, Hideki Hirata, Hiroshi Honda

**Affiliations:** 1 Department of Clinical Radiology, Graduate School of Medical Sciences, Kyushu UniversityFukuokaJapan; 2 Department of Radiology, Fukuoka University School of MedicineFukuokaJapan; 3 Department of Radiologic Technology, School of Health Sciences, Kyushu UniversityFukuokaJapan

## Abstract

**Introduction:**

Primary malignant melanoma of the esophagus is a rare but highly aggressive tumor with poor prognosis. Surgical resection is the treatment of choice. However, some cases may be diagnosed with advanced inoperable disease. Palliative radiotherapy may be used to relieve symptoms caused by the esophageal tumor.

**Case presentation:**

We report on a case of advanced inoperable primary malignant melanoma of the esophagus treated with palliative radiotherapy. The patient’s dysphagia resolved with radiotherapy.

**Conclusion:**

Malignant melanoma of the esophagus is rare. Patients with advanced inoperable malignant melanomas of the esophagus benefit from radiation therapy. Radiation therapy is effective for palliation.

## Introduction

Primary malignant melanoma of the esophagus is an uncommon disease accounting for only 0.1 % to 0.5 % of all esophageal carcinomas [1-3]. It has a highly aggressive tumor behavior with a median survival of about 10 months [4]. Some patients are inoperable at presentation. Radiation therapy has been used to relieve symptoms. Malignant melanomas are generally considered radioresistant, requiring higher dose per fraction. We report on a case of primary malignant melanoma of the esophagus treated with palliative radiotherapy.

## Case presentation

**Figure 1.**
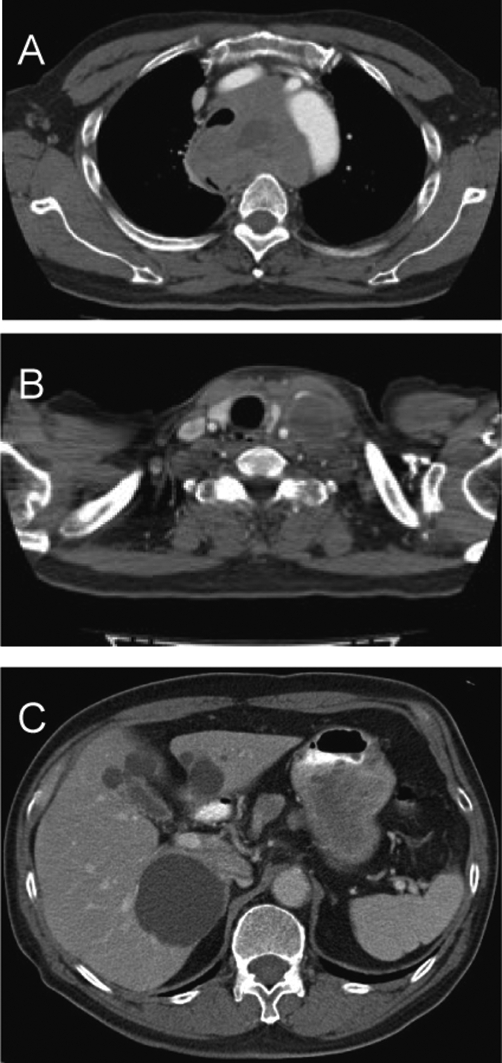
Contrast enhanced CT showed 7.7 cms mediastinal lymph nodes compressing the esophagus and trachea at the level of the aortic arch **(A)**, 3.3 × 3.0 cms left supraclavicular **(B)** and 1.9 × 1.5 cms abdominal lymph nodes **(C)**.

**Figure 2.**
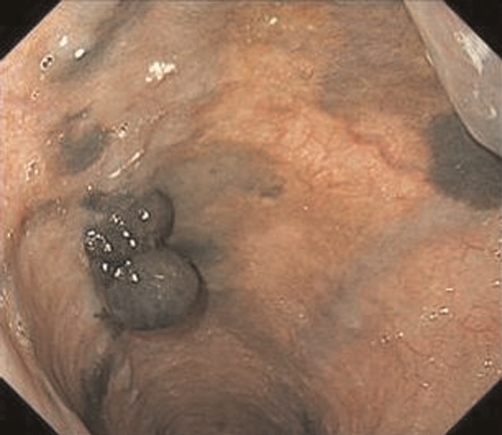
Endoscopic view of a dark gray polypoidal tumor of the esophagus at presentation.

A 71-year-old male subject presented to us in very poor general condition (Eastern Cooperative Oncology Group performance status 3) with a history of hoarseness (3 months) and dysphagia (1 month). Clinically, the left supraclavicular lymph node (3 × 3 cms) was palpable. Computed tomography (CT) scan of the chest and abdomen demonstrated extensive swelling of the mediastinal lymph nodes extending from 4 cm above the level of the upper margin of the sternum to 2 cm below the carina, the esophagus was compressed and trachea shifted to the right of the midline. Primary esophageal tumor was not separately identifiable from the mediastinal lymph nodes. Lymph nodes in the superior and middle mediastinum measured approximately 12 × 7.7 × 6.0 cm (Figure 1A). Left supraclavicular (3.3 × 3.0 cm) and abdominal node (1.9 × 1.5 cm) metastases were also observed (Figure 1B,1C). Esophagoscopy demonstrated a 10 mm pigmented polypoidal lesion (25 cm from the inscisor) and multiple pigmented flat lesions (Figure 2). Esophageal narrowing due to extrinsic compression was seen from 19 cm to 24 cm. Histopathology of the biopsy specimen from the esophagus showed diffuse proliferation of atypical polygonal cells with hyperchromatic oval to round nuclei, eosinophilic cytoplasm, and prominent nucleoli, accompanied by various amounts of melanin pigments and immunohistochemically - positive for S-100 protein. These findings were all compatible with malignant melanoma of the esophagus. A final diagnosis malignant melanoma of the esophagus T4N1M1a, stage IVa (UICC-TNM classification [5]) was made.

**Figure 3.**
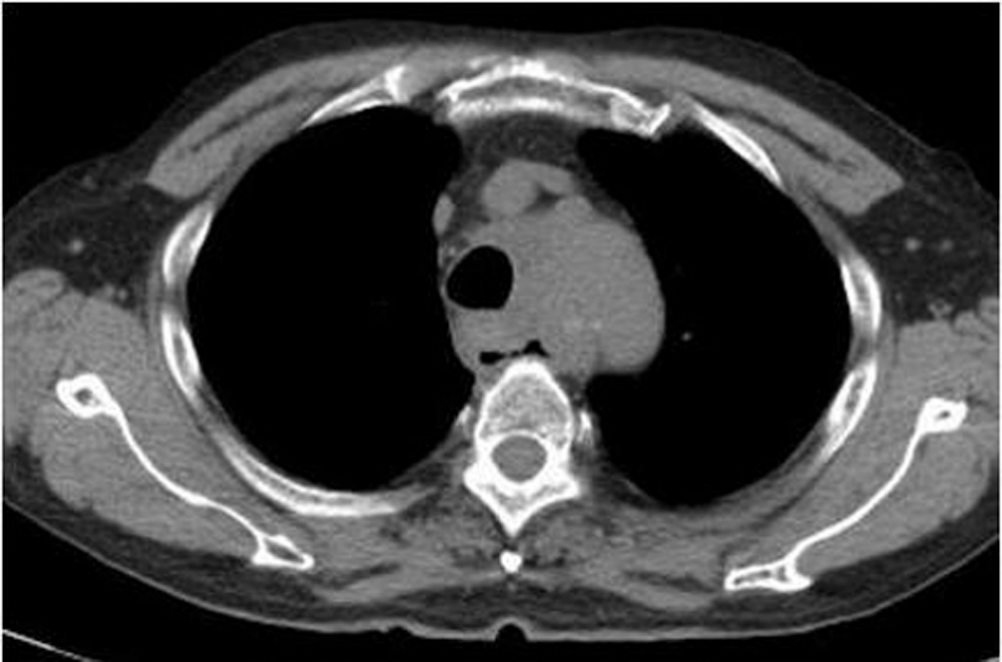
Mediastinal lymph node decreased in size on plain CT 1 week following completion of radiotherapy at the level of the aortic arch.

Due to the fact that PS was 3, palliative radiotherapy was offered for palliation of dysphagia and tracheal compression and chemotherapy was not administrated. He received radiation therapy for primary tumor and mediastinal and left supraclavicular lymph nodes in a dose of 45 Gy (gray) in 15 fractions over 3weeks at 5 fractions per week, using anteroposterior and posteroanterior field with10-MV photon. Patient tolerated the radiotherapy well. The patient’s dysphagia resolved completely shortly after radiotherapy. 1 week after radiotherapy there was a marked decrease in the size of the mediastinal lymph nodes and subject improved symptomatically. The axial dimensions of the lymph node were maximal around the level of the aortic arch where it measured approximately 4.5 × 3.5 cm 1 week following completion of radiotherapy (Figure 3). Patient had sustained palliation i.e, continuous relief of dysphagia for 4 months after radiotherapy.

## Discussion

Primary malignant melanoma affecting the esophagus is a rare and fatal disease with a poor prognosis. Sabanathan *et al.* documented that approximately 50% of patients have metastatic disease at presentation and long-term survival is extremely rare [6]. The clinical presentation of primary malignant melanoma of the esophagus is similar to the common forms of primary esophageal malignancies. The tumor most commonly occurs in the 6^th^ and 7^th^ decade, and the male to female ratio is 2:1. Subtotal esophagectomy is recommended as the treatment of first choice for primary malignant melanoma of the esophagus. Sabanathan *et al.* reported a median survival of between 7 and 12 months in patients who underwent radical resection and a 5 year survival of 4.2 % [7]. Chalkiadakis *et al.* reported, in a series of 110 patients, a mean survival of 13 months [8]. However, at the time of diagnosis, some cases may be diagnosed as having inoperable carcinoma. Chemotherapy is one of the treatment options for malignant melanoma. However, it is difficult to use chemotherapy to elderly or poor performance status patients. Paul *et al.* reported that the response rate for dacarbazine alone was 16.9 % and was 21.5% for dacarbazine plus immunotherapy [9]. Although radiotherapy may have a palliative role if surgery is not possible, malignant melanoma has been considered radioresistant [10]. At the time of diagnosis of the present case, multiple lymph node metastases were observed and the performance status was 3. Therefore, radical surgery and chemotherapy were not considered. There have been several case reports of effective palliation with photon radiotherapy [11,12]. Forgarty *et al.* reported that a patient with malignant melanoma of the esophagus received 36 Gy with a photon beam in six fractions given twice weekly, and CT after 4 months following completion of radiotherapy showed no residual mass [11]. In the present case, palliative radiotherapy using a once-daily fractionation of 3.0 Gy was offered, and the tumor markedly decreased in size and partial response was achieved.

## Conclusion

Malignant melanoma of the esophagus is rare. Patients with advanced inoperable melanomas of the esophagus benefit from radiation therapy. Radiation therapy is effective for palliation.
